# Acupuncture treatment for carotid atherosclerotic plaques: study protocol for a pilot randomized, single blinded, controlled clinical trial

**DOI:** 10.1186/s13063-020-04709-0

**Published:** 2020-09-07

**Authors:** Junhe Zhou, Lin Zhao, Lingcui Meng, Huitao Liang, Ting Zhou, Siting Ye, Zhiqi Qi, Xichang Huang, Peng Zhou, Wenbin Fu

**Affiliations:** 1 The Second Affiliated Hospital of Guangzhou University of Chinese Medicine, Guangzhou, Guangdong Province China; 2 The Second Clinical College, Guangzhou University of Chinese Medicine, Guangzhou, Guangdong Province China; 3 Ultrasonography Department, The Second Affiliated Hospital of Guangzhou University of Chinese Medicine, Guangzhou, Guangdong Province China; 4 Acupuncture Department, The Second Affiliated Hospital of Guangzhou University of Chinese Medicine, Guangzhou, Guangdong Province China; 5 Shenzhen Bao’an Research Centre for Acupuncture and Moxibustion, Shenzhen, China

**Keywords:** Carotid atherosclerosis disease, Cervical atherosclerosis plaque, Acupuncture, Vessel plaque quantification (VPQ)

## Abstract

**Background:**

Carotid atherosclerosis disease (CAD) is generally associated with the occurrence of cardiovascular and cerebrovascular accidents. However, CAD has not been taken seriously enough in the clinic, which, coupled with the single treatment and prevention of CAD, has led to a generally low level of patient compliance. Therefore, acupuncture is expected to be a safe and effective therapy that can be maintained in the long term for patients with CAD. The study objective is to evaluate the efficiency and reliability of acupuncture to relieve CAD and provide a new therapeutic idea for the clinical treatment of CAD.

**Methods:**

This is a three-arm randomized clinical trial in China. Three groups (TA, SA, and MC) will be randomly allocated at a 1:1:1 ratio. The study will enrol 105 cervical atherosclerosis plaque patients in total on a voluntary basis, with 35 patients in each group. The treatment will last for 12 weeks, with two treatments per week for twenty-four treatments in total.

**Results:**

Two 3D ultrasound indicators will be measured as the primary outcomes: the total plaque volume (PV) of the carotid artery on each side and the grey-scale median (GSM). The secondary outcomes will include intima-media thickness (IMT), lipid levels, apolipoprotein A-IV level, platelet count (PLT), fibrinogen (FIB), and platelet aggregation rate (PAR). All the outcomes will be assessed before treatment, after treatment, and after a 12-week follow-up period. This study will utilize per-protocol (PP) and intention-to-treat (ITT) analysis principles.

**Conclusions:**

This trial is to evaluate the efficacy and reliability of acupuncture in relieving carotid atherosclerotic plaques by establishing acupuncture (TA), sham acupuncture (SA), and medication (MC) groups.

**Ethics and dissemination:**

This study was approved by the Institutional Ethics Committee of Guangdong Provincial Hospital of Traditional Chinese Medicine (no. YF2018-107-01). All data and findings will be provided by the principal investigator via email.

**Trial registration:**

ChiCTR, ChiCTR1800019259. Registered on 1 November 2018—retrospectively registered, http://www.chictr.org.cn/index.aspx

## Background

Atherosclerotic disease is a type of disease associated with arthritis, lipids, and other metabolic disorders in the body. Atherosclerotic disease is the main cause of cardiovascular and cerebrovascular diseases. According to research, in 2013 alone, 247.9 people died per 100,000 people due to these diseases, accounting for 28.2% of the total number of deaths [1]. Although the population structure has changed, the mortality rate of cardiovascular and cerebrovascular diseases has decreased by 22% [2]. A study found that one American dies every 1 min and 23 s from this type of disease, and a coronary event occurs every 34 s [3]. A 2009 study reported that the indirect and direct costs of cardiovascular disease and stroke in the USA were as high as $31 billion [4]. A study has counted the disease burden in China from 1990 to 2010. Among them, the main cause of death was stroke, and 1.7 million patients died of stroke in 2010. Ischaemic heart disease ranks second, with 948,700 cases of death from ischaemic heart disease, which both have intricate interactions with atherosclerosis [5].

For treatment, antiplatelet drugs and statins are often used in the clinic. Both antiplatelet drugs and statins can effectively reduce the incidence of cardiovascular and cerebrovascular diseases [6–9]. Additionally, aspirin combined with statins can reduce the incidence of cardiovascular events in patients with diabetes [10].

However, studies have found that antiplatelet drugs are not suitable for drug absorption and can cause gastrointestinal irritation and bleeding [11–13]. A survey found that 7–29% of patients taking statins developed myositis [14].

In a multi-centre study that included 2864 participants, the incidence of intracranial stenoses in patients with acute ischaemic stroke was 46.6%, of which 9.11% of cases were associated with carotid stenosis. Stroke patients with head and neck artery stenosis have more severe symptoms after admission and a higher prevalence of recurrent strokes. Head and neck arterial plaque should be an important intervention target for primary prevention before stroke [15].

Acupuncture is used to improve diseases caused by atherosclerosis; for example, acupuncture is used for rehabilitation after stroke. The curative effects of acupuncture include improved blood flow in the ischaemic area after stroke, anti-apoptosis, and the promotion of the growth of nerve cells. For example, acupuncture may promote the proliferation of neural stem/progenitor cells in response to ischaemia via the Wnt/β-catenin pathway and by modulating retinoic acid expression [16–18]. Recent studies have found that acupuncture relieves angina. The acupuncture points for angina pectoris are bilateral acupoints PC6 and HT5 [19]. Acupuncturists often choose BAIHUI (DU20), YINTANG (EX-HN3), and YANGLINGQUAN (GB34) to treat stroke. Research on the efficacy and mechanism of acupuncture in improving atherosclerotic plaque is still insufficient. Acupuncture is widely used in the rehabilitation treatment of atherosclerotic diseases, but acupuncture is mostly used after cardiovascular and cerebrovascular events occur. The purpose of this study is to provide data support for acupuncture in the treatment of atherosclerotic plaques and to provide a new idea for the early prevention of atherosclerosis.

The main objective is to evaluate the efficiency and reliability of acupuncture to relieve carotid atherosclerotic plaques by establishing TA, SA, and MC groups.

## Methods

### Study design

**Fig. 1:**
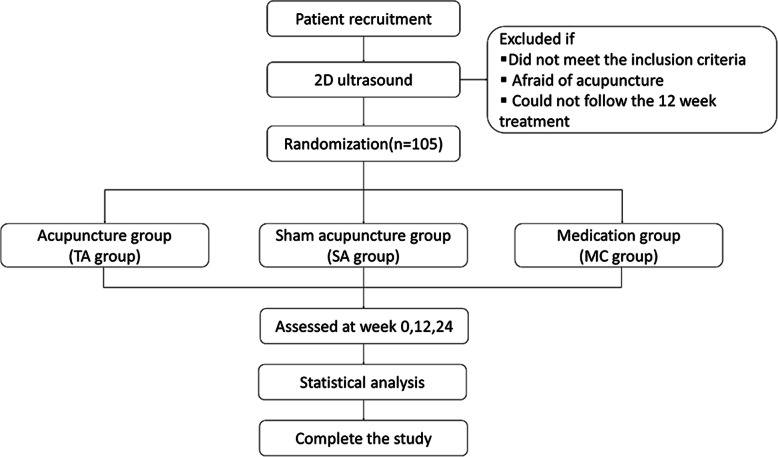
Diagram of trial design, including patient recruitment, inclusion criteria, randomization, three groups (TA group, SA group, MC group), assessment timepoints, and statistical analysis

This is a three-arm randomized, parallel control clinical trial. The study objective is to evaluate the efficiency and reliability of acupuncture to relieve CAD. Three groups will be randomly established at a 1:1:1 ratio, including the TA group (receiving acupuncture therapy), the SA group (receiving sham acupuncture therapy), and the medication group (taking aspirin and statins). The study will enrol 105 individuals in total, 35 individuals in each group. The medication group is set for the positive control to evaluate the difference in the curative effect between acupuncture and drugs. At the same time, sham acupuncture group is set for placebo control to evaluate the clinical efficacy of acupuncture. The flowchart of this protocol can be found in Fig. 1.

### Inclusion criteria

- Conformation to the diagnostic criteria according to the Guidelines for Diagnosis and Treatment of Head and Neck Arteriosclerosis in China (2017) [20];
- No cardio-cerebrovascular diseases history;
- No vulnerable plaque and plaques on both sides that amount to less than five plaques;
- Cholesterol from 2.59 to 8.0 mmol/L and triglycerides (TGs) from 1.7 to 10.0 mmol/L;
- Age from 25 to 70 years old (unrestricted sex);
- No use of any medicine for atherosclerosis during the last 2 weeks;
- Agreement to participate the study and signed the informed consent before inclusion.

### Exclusion criteria

- Carotid aortic stenosis greater than 50% on any side;
- Greater than or equal to five plaques on both sides;
- Body mass index (BMI) ≥ 30; hyperlipidaemia patients with cholesterol greater than 8.00 mmol/L. Triglyceride is greater than 10.0 mmol/L;
- A history of stroke, myocardial infarction (MI), craniocerebral injury, major surgery, arrhythmia, angina, coagulopathy, thyroid disease, liver and kidney insufficiency, psychiatric disorders, or drug abuse;
- Presence of more than 4 of the following risk factors: older than 60 years, hypertension, smoking, diabetes, obesity, and low high-density lipoprotein (HDL).

### Dropout criteria

- Occurrence of severe cardiovascular and cerebrovascular disease or complications which result in stopping the trial;
- Patients who have severe adverse reactions after acupuncture and cannot successfully complete the course of treatment;
- The participant was unable to follow the protocol treatment for personal reasons during the course, or reject to continue the study.

### Study setting

**Fig. 2:**
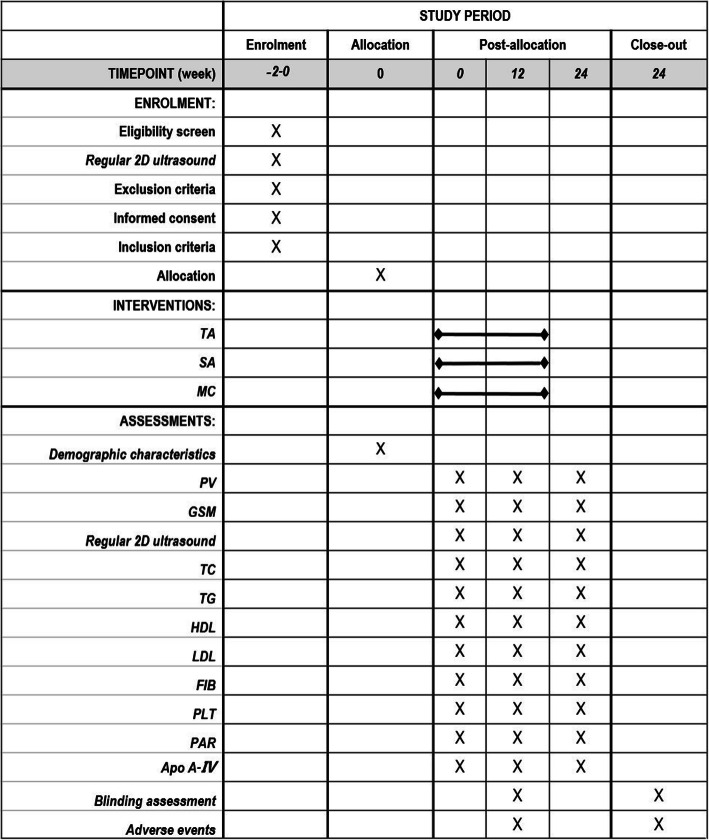
SPIRIT figure of enrolment, interventions, and assessments. TA, the acupuncture group; MC, the medication group; SA, the sham acupuncture group; PV, the total plaque volume; GSM, the grey-scale median; APO A-IV, apolipoprotein A-IV; PLT, platelet count; FIB, fibrinogen; PAR, platelet aggregation rate; LDL, low-density lipoprotein; TG, triglyceride; HDL, high-density lipoprotein

The study team will advertise to the public via the advertising department of the Guangdong Provincial Hospital of Chinese Medicine. The treatment will be conducted at the acupuncture clinic of Guangdong Provincial Hospital of Chinese Medicine. The study will enrol 105 individuals in total, with 35 individuals in each group. Patients who are included in the study are required to sign an informed consent form and will participate in the study on a voluntary basis. The schedule of patient enrolment, intervention, and assessment is illustrated in Fig. 2.

### Recruitment

We will recruit the participants in the physical examination, cardiovascular, and acupuncture departments of the four branches in Guangzhou. At the same time, participants will be recruited through the Guangzhou Volunteer Recruitment Network Platform.

The team staffs will give proper healthy dietary guidance to all participants during after the whole study period to promote subject retention and complete follow-up, as well as reminding the participants of their treatment or assessment schedule. WeChat group will be forming to all participants to communicate with doctors and report adverse events at any time.

### Randomization

Baseline data of participants will be collected within 2 weeks and evaluated before random assignment. Participants will be assigned to the TA group, the MC group, or the SA group at a ratio of 1:1:1. To ensure the equality of random distribution, the generation of random sequences will be performed by the Acupuncture Research Institute, who will not participate in this study. Random numbers and grouping results will be saved in opaque envelopes by the staff who performed the randomization. All envelopes will be kept by staff who will not participate in the study. The research assistant will open the envelopes to assign them in the order in which they are registered.

### Blinding

Because of the characteristics of acupuncture, the acupuncturists in this study cannot be blinded to the assignment, but the participants can be blinded. The treatment, testing, and follow-up of each group will be implemented independently, with no intersection between each group. The sham acupuncture in this study can guarantee a painful effect, similar to acupuncture, but does not penetrate the skin; sham acupuncture will allow for the better blinding of the participants. In addition, participants in the TA and SA groups will be asked three questions at the end of the 12 weeks to test the blinding effect: ‘Do you have confidence in this treatment?’ ‘Would you recommend this therapy to your friends?’ ‘Can you accept this kind of acupuncture?’ The participants will choose ‘Yes’ or ‘No’ to answer the questions. The data will be analysed at the end of the study. If the results are not significant, then it can be concluded that the blinding was sufficient. To ensure the uniformity of ultrasound examination and blood testing, all ultrasound examinations will be completed by a doctor with over 10 years of vascular ultrasound work experience. The Department of Clinical Laboratory of Guangdong Provincial Hospital of Traditional Chinese Medicine is responsible for the whole blood testing process. Both the ultrasound doctor and clinical laboratory will be blinded to the assignment. Collectors and statisticians responsible for collecting data and statistics will be blinded to the assignment.

## Intervention

**Fig. 3:**
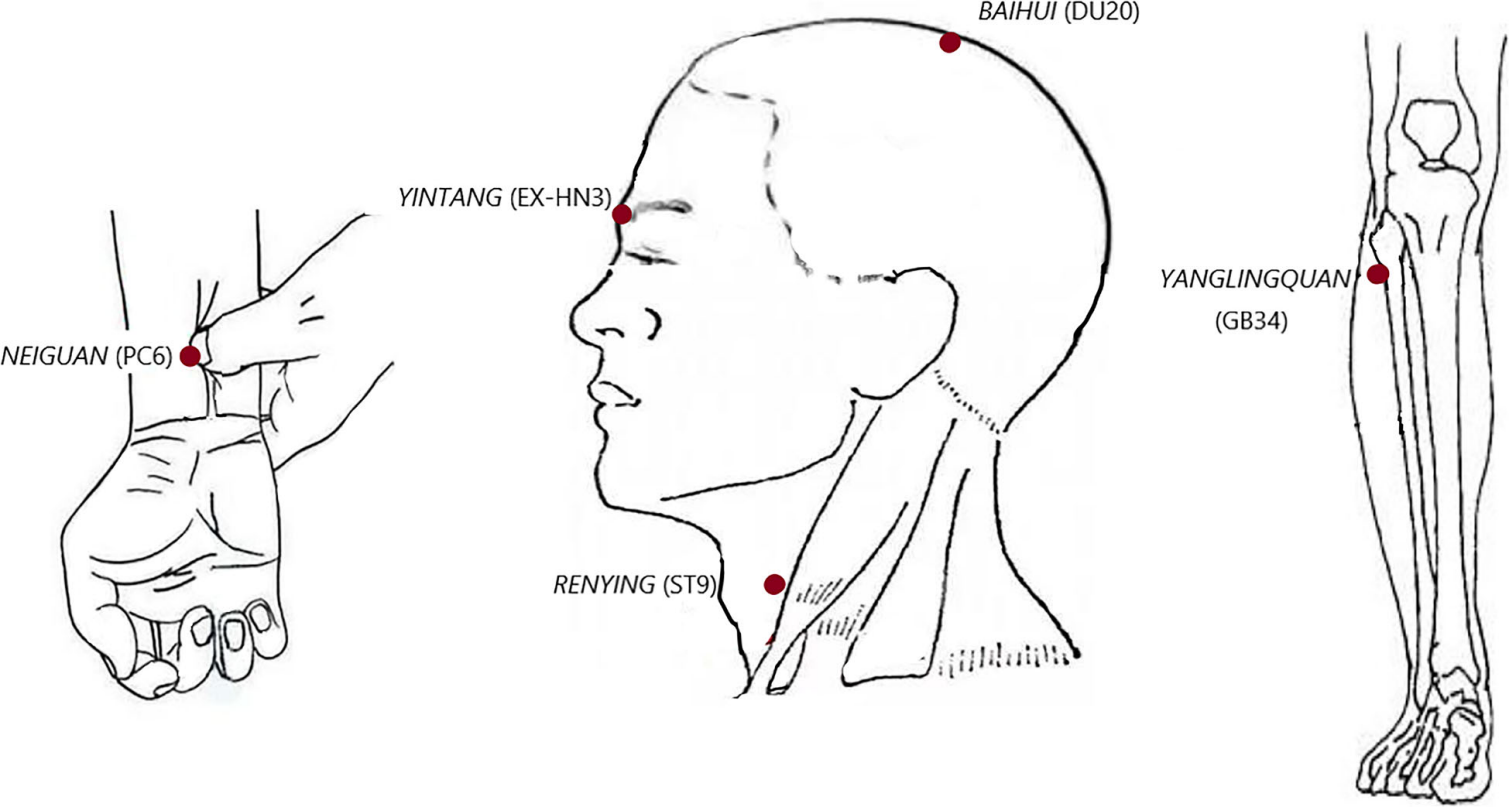
Acupoint diagram of the protocol. The eight acupoints used in the protocol visually are shown. NEIGUAN (PC6), RENYING (ST9) (both sides), BAIHUI (DU20), YINTANG (EX-HN3), and YANGLINGQUAN (GB34) (both sides) are applied in total. The specific location of these acupoints is listed in Table 1

The protocol design was based on previous clinical research and the classic theory of Chinese medicine. All operations adhered to the Standards for Reporting Interventions in Clinical Trials of Acupuncture (STRICTA) standard [21]. Acupoints applied in the RA and SA groups are the same as those listed in Table 1 and Fig. 3, according to the WHO Standard Acupuncture Locations guideline [22]. All acupuncturists who will take part in the research will be qualified to perform acupuncture with at least 5 years of study experience or 3 years of related work experience. Before implementation, all the researchers engaged in this study will participate in a unified training programme together. The groups will undergo the treatments separately twice per week for 24 treatments in total. In order to ensure the rights and interests of the patients and meet the ethical requirements, the subjects are allowed to use the treatment for diseases (such as cervical spondylosis, pain, insomnia, and fever) which are not closely related to atherosclerosis during the study period. It should be noted that the treatment received by the patients cannot interact with the drugs taken in the study, and the patients cannot receive any other acupuncture treatments so as not to interfere with the evaluation of the efficacy of the interventions included in the study.

**Table 1:** Acupoints in both the TA group and the SA group

Name	Locations
*NEIGUAN* (PC6)	On the palmar side of the forearm, 2 in. superior to the transverse crease of the wrist, between the palmaris longus tendon and the radial wrist flexor tendon.
*RENYING* (ST9)	Lateral to the laryngeal prominence of the cervical region, the anterior margin of the sternocleidomastoid, at the fluctuation place of the common carotid artery.
*BAIHUI* (DU20)	On the median line of the head, 5 in. superior to the anterior hairline, at about the middle of the connecting line between both auricular tips.
*YINTANG* (EX-HN3)	At the middle between the two eyebrows on the forehead.
*YANGLINGQUAN* (GB34)	On the lateral side of the shank, in the depression anterior and inferior to the head of the fibula.

### TA group

Before undergoing acupuncture, patients will be asked to lie in the supine position in a quiet environment. First and foremost, the acupuncturist will sterilize his or her fingers and the skin near the acupoints with 0.5% iodine. Then, quick needle insertion will be performed with a stainless steel needle (30 × 25 mm, Huatuo, Suzhou, China) by skilled acupuncturists. The depth of insertion is flexible and depends on the location of the acupoints and the figure of the patient. NEIGUAN (PC6), YANGLINGQUAN (GB34), and RENYING (ST9) will be inserted first, while BAIHUI (DU20) and YINTANG (EX-HN3) will be inserted subsequently. After that, the needle will be twirled gently and retained for 30 min after the arrival of Qi. When withdrawing the needles, acupuncturists will be asked to press the pinholes with cotton swabs for 2 min in case of bleeding, especially for the RENYING (ST9) acupoint.

### SA group

**Fig. 4:**
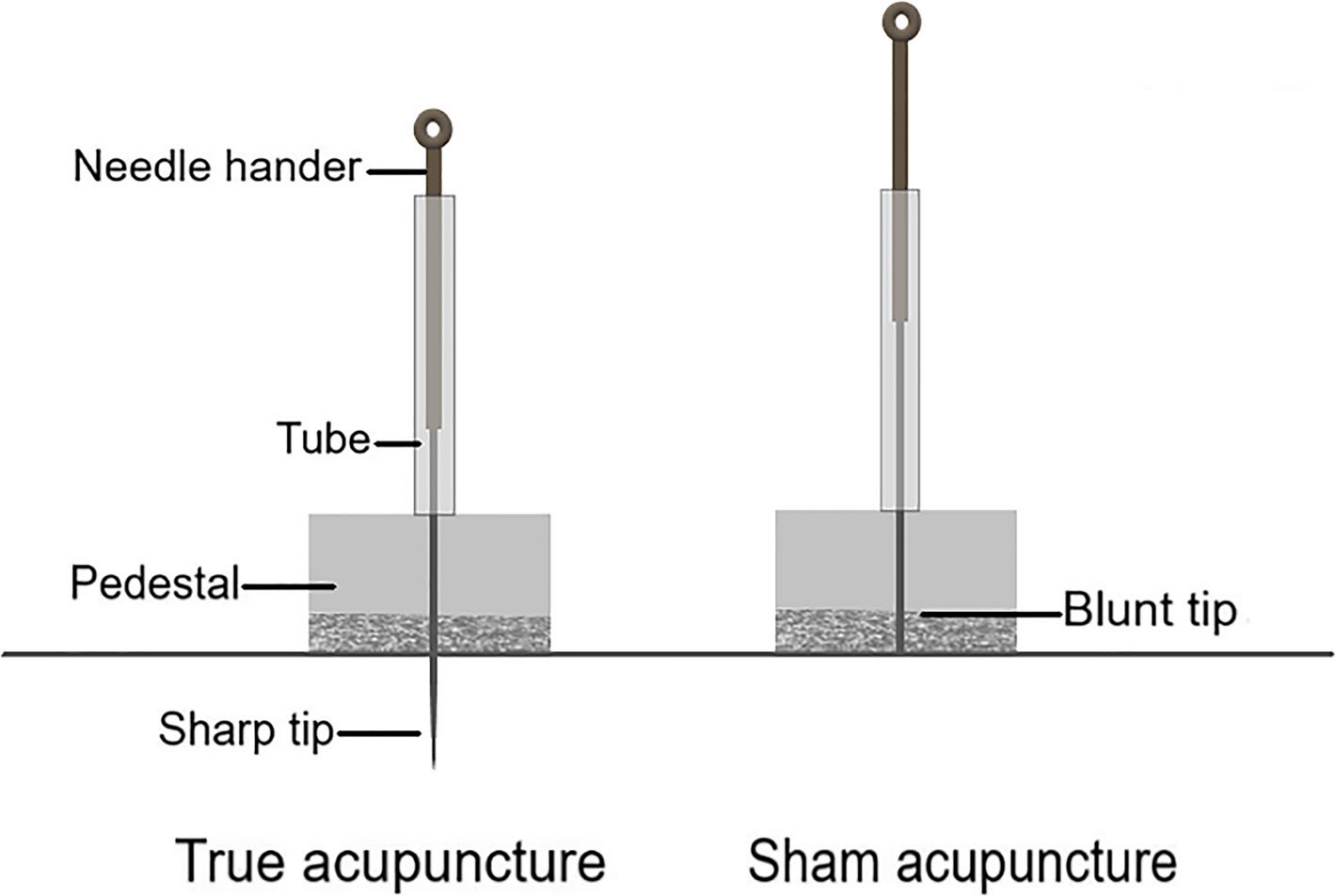
The operation of sham acupuncture. The sham acupuncture model is made up of the translucent tube, the double-sided adhesive, and the blunt needle which can keep the needle standing vertically without piercing into the skin

The environmental requirements, the acupuncture position, and the treatment frequency of the SA group will be consistent with those of the TA group. The protocol uses a specific blunt needle (30 × 25 mm, Huatuo, Suzhou, China). Acupuncturists will immobilize the translucent tube with double-sided adhesive on the same acupoints as those used in the TA group; then, the acupuncturists will pretend to insert the blunt needle through the tube and keep the needle standing vertically. The patients will feel a little stabbing pain; however, they are not likely to know that the needle did not prick their skin. The operational difference between TA and SA is shown as follows in Fig. 4.

### MC group

Patients randomized to the MC group will be given aspirin enteric-coated tablets (100 mg qd po, Bayer) and atorvastatin calcium tablets (10 mg qd po, Pfizer) for antiplatelet aggregation as well as lipid-lowering effects. The drugs will be administered and provided to patients for free every week by the researchers. Once the patients forget to take pills or stop for personal reasons, researchers will record the detailed to individual situations. If patients are required to take other drugs for a long period due to illness, then the drug name, dosage, and frequency will be recorded.

## Outcome

Ultrasound and biochemical indicators will be used as outcome measures. The primary outcome consists of the total PV and the grey-scale median (GSM), and intima-media thickness (IMT), lipid levels, apolipoprotein A-IV level, platelet count (PLT), fibrinogen (FIB), and platelet aggregation rate (PAR) are the secondary outcomes. Biological specimens will be stored at − 80 °C after collection. All the biological specimens will be analysed in the current trial and for future use in ancillary studies.

### Primary outcome

Vessel plaque quantification (VPQ) is preferred for observing plaque morphological characteristics as a non-invasive assessment method [23]. The vessel plaque quantification technique has a substantial advantage over traditional two-dimensional ultrasound, which has been used for vascular event risk prediction. There are two aspects to be measured by the same sonologist: the total PV of the carotid artery on each side and the grey-scale median (GSM), which can indicate the average hardness of plaques proportionally [24, 25]. Both the PV and GSM will be assessed on weeks 0, 12, and 24 on each side.

### Secondary outcomes

#### Regular carotid ultrasound

For diagnosing atherosclerosis, every participant will be required to undergo regular carotid ultrasound for screening before entry. Intima-media thickness (IMT) will be measured at study entry, exit, and follow-up. A prior study has documented that people with higher IMT are more likely to suffer from cardiovascular disease (CVD), especially stroke [26]. Aside from IMT, the maximum thickness of a single plaque, which is calculated by Crouse integration, will be measured throughout the study at each assessment.

#### Lipid level testing

As a previous study described, dyslipidaemia, such as high TGs and high total cholesterol (TC), usually occurs along with atherosclerotic plaques. Therefore, the modification of blood lipids, including TGs, TC, HDL, and LDL, seems to play a vital role in controlling the progression of atherosclerosis. Patients will undergo three blood tests to estimate their lipid level before and after the treatment, as well as after the follow-up period. The blood sample collection will be performed with the participants in a fasting state before 10 am.

#### Apolipoprotein A-IV testing

Apolipoprotein A-IV is a type of HDL that can activate LCAT and participate in the antidromic transport of TGs. Prior to the work of Xu et al. [27], apolipoprotein A-IV was considered a new-found ligand of αIIbβ3 integrin on platelets. In other words, apolipoprotein A-IV can antagonize the combination between platelets and fibrinogen. Consequently, the study will use apolipoprotein A-IV as one of the secondary outcomes to determine whether acupuncture could inhibit platelet aggregation by improving apolipoprotein A-IV levels in the blood.

#### Coagulation testing

Several coagulation-related measures are also involved in this protocol; these measures consist of platelet count (PLT), fibrinogen (FIB), and PAR. Thus far, platelet aggregation and hypercoagulability are regarded as the initial factors of atherothrombosis that can ultimately cause myocardial infarction or cerebrovascular accidents.

### Sample size

This is a three-arm study. In our search for previous studies of the effects of acupuncture on the carotid atherosclerotic plaque, it was found that most of the studies were two-arm studies of acupuncture versus drugs. There is a lack of high-quality clinical research. In addition, the effect of acupuncture exhibits a significant correlation between acupoints. Therefore, it is not appropriate to use the previous acupuncture clinical research literature data to estimate the sample size. Furthermore, this is a pilot study to guide future randomized controlled trials and to test the feasibility of the protocol. Considering that there are many clinical blood tests in the study, we will choose the clinical minimum sample size of 30 individuals for each group to reduce the burden of detection of the subjects. The maximum dropout rate within the intervention is expected to be approximately 15%. The total number of patients needed to be randomized is therefore 105 (35 for each group).

### Safety assessment

To reduce the risk of adverse events, firstly, acupuncturists should have years of work experience. Before the study begins, all acupuncturists need a unified training to standardize the acupuncture operation and increase the safety of acupuncture. Secondly, during the research process, the cardio-cerebrovascular doctor will evaluate the patient’s condition to ensure that the patients with severe cardio-cerebrovascular disease during the study period can be treated in time. During the treatment, the acupuncturist will also evaluate the subject’s condition to reduce the occurrence of adverse events. Finally, this study will be conducted in Guangdong Provincial Hospital of Traditional Chinese Medicine. The hospital has a strong medical level and ability to handle crisis events, which can guarantee the progress of this study. Acupuncture may cause some adverse reactions, such as bleeding, redness, fainting, and pain. All adverse events need to be recorded in common reporting format (CRF) tables. Adverse event records should include time, symptoms, extent, and their relationship with acupuncture. The investigator should handle and record adverse events. Serious adverse events should be reported to the Guangdong Provincial Hospital Ethics Committee. Participants who are unable to continue participating in the study due to adverse events will be excluded.

### Data collection and management

This study will collect population data from eligible participants before the first treatment; these data include name, age, duration, occupation, blood pressure, sex, and body mass index (BMI). Drug and acupuncture adverse reactions will be recorded during the treatment and follow-up periods based on the reaction of the participants. At weeks 0, 12, and 24, data on carotid ultrasound, blood lipids, platelet aggregation, fibrinogen, platelet count, and apolipoprotein A-IV will be collected. The data collection will be performed by three staff members who will not take part in acupuncture treatment, index evaluation, statistical analysis, or grouping. The data collection and entry will be performed independently by two of the staff members and finalized by a third staff member. The main investigators, acupuncturist, sonographers, and laboratory staff will not be involved in data collection.

All data must be saved in paper with identification codes in seal, while electronic data is saved on the ResMan Research Manager of the Clinical Trial Management Public Platform, and photo formats are also named with identification codes to ensure authenticity and integrity. All data must be saved in paper, electronic, and photo formats to ensure authenticity and integrity. The data should be kept for at least 5 years after the article is published. A data monitoring committee was also set up in this study, composed of experts with good clinical research experience in the Department of Acupuncture of Guangdong Provincial Hospital of Traditional Chinese Medicine. Experts will regularly monitor research progress, data, participant management, distribution, and more. The committee is independent from the fund sponsor and has no conflicts of interest. Researchers who take charge in the data collection and entry will have access to the interim results and report to main investigators if necessary. Consequently, the main investigators will make the final decision to terminate the trial after discussion.

### Quality control

Before the study, all researchers will be required to accept uniform training. Researchers should be fully aware of the purpose and composition of this study. All acupuncturists will be required to have obtained a licence and have worked for more than 3 years. Before the study, acupuncturists will be trained to clarify the acupuncture standard in this study. During the study, the main investigator will check the CRF every week. The grouping, data collation, and data analysis will be carried out by specific staff members, and there will be no work intersect between these members. The adverse events that might occur during the study will be recorded in detail. The participants’ personal data will be kept by the researcher, and no one other than the researcher is allowed to access the data.

### Statistical analysis

This study will use per-protocol (PP) and intention-to-treat (ITT) analysis principles. ITT will include all subjects who participated in at least one treatment, including participants who dropped out. Missing data for participants who dropped out will be supplemented with the most recent data. The PP principle will analyse all participants’ data that conform to the study protocol, and those of individuals who did not conform to the protocol will not be included.

The data will be analysed by professional statisticians with SPSS 20.0 software (IBM SPSS Statistics, IBM Corp, Somers, NY, USA). First, we will describe the baseline data, such as age, blood pressure, course of the disease, BMI, and sex, and compare the baseline data between the three groups. The continuous variables will be presented as the mean ± standard deviation (SD), and the categorical variables will be presented as the composition ratio and rate.

Continuous variables that conform to a normal distribution will be analysed by ANOVA, and the non-conforming data will be analysed using the rank sum test. Categorical variables will be analysed using the chi-square (*χ*^2^) test or Fisher’s exact test. The confidence interval (CI) will be estimated at 95%, and the significance level will be set at 0.05. A *P* value < 0.05 will be considered statistically significant. The adverse data will be recorded and analysed for the relationship with acupuncture. We will calculate the dropout rate and analyse the reasons for dropping out.

### Ethics and dissemination

The study was planned in accordance with the Declaration of Helsinki. This study was approved by the Institutional Ethics Committee of Guangdong Provincial Hospital of Traditional Chinese Medicine (approval no. YF2018-107-01). Trial has been registered at the Chinese Clinical Trial Registry (ChiCTR1800019259). If there is any change to the clinical research protocol and informed consent, the researchers will be asked to report the modifications to both the ethics committee and registry centre timely.

Before the participants are enrolled in the study, the researchers will fully inform the patient of the treatment and tests that need to be completed and fully inform the participant regarding the risks that may exist in the study and their power to withdraw from the study. Patients are also informed enough to understand the possible adverse reactions of collecting biological specimens. All treatments and tests in this study will be provided for free. Throughout the research process, we will provide free medical advice and guidance to all participants. After all follow-ups, we will provide 3 months of free acupuncture treatment for participants in the SA group. The participants will also be paid for part of their travel expenses to and from the hospital, all of which will be offered to enhance participant compliance.

### Patients and public involvement

An acupuncture patient originally proposed acupuncture treatment for carotid atherosclerotic plaque. None of the other participants in the study participated in the design or evaluation. The burden of the intervention will be assessed by the patients themselves. The results will be disseminated to study participants via email or phone messages.

### Trial status

The trial is currently in the recruitment phase. The protocol is registered on November 1, 2018. The protocol number is ChiCTR1800019259. The recruitment started on July 1, 2018, and as of October 2019, a total of 42 people had participated in the study. The expected date of trial completion is June 30, 2020.

## Discussion

The rupture of carotid atherosclerotic plaque is one of the risk factors for cerebrovascular disease, and a clinical emphasis has been placed on the early treatment and prevention of cardiovascular events [28]. Although clinical antiplatelet aggregation and lipid-lowering drugs have been widely used, the side effects of drugs are still difficult to avoid. Therefore, we hope this pilot study could provide an effective data basis for subsequent large-sample studies of acupuncture treatment for atherosclerotic plaque.

### Comprehensive selection of outcome measures

Doppler ultrasound technology is an economical, simple, non-invasive detection method that is widely used for carotid atherosclerotic plaque and stenosis detection. The vessel plaque quantification technique used in this study can assess PV, plaque composition, and plaque remodelling structure.

The GSM value is an average evaluation of the plaque on one side of the carotid artery, which can effectively evaluate the components of the plaque. The lower the GSM value, the higher the lipid content in the plaque, and the higher the GSM value, the higher the calcium and fibre content in the plaque [24]. The GSM value of the plaque is related to plaque stability, and plaques with high lipid content usually have higher instability.

Lipid metabolism plays a key role in plaque elimination. HDL has anti-atherosclerosis effects, and its concentration is negatively correlated with the occurrence and severity of atherosclerosis. The main component of HDL is apolipoprotein A-I (70%), followed by apolipoprotein A-II and apolipoprotein A-IV. In this study, we will observe whether acupuncture plays an anti-atherosclerotic plaque role by regulating blood lipid levels.

**Fig. 5:**
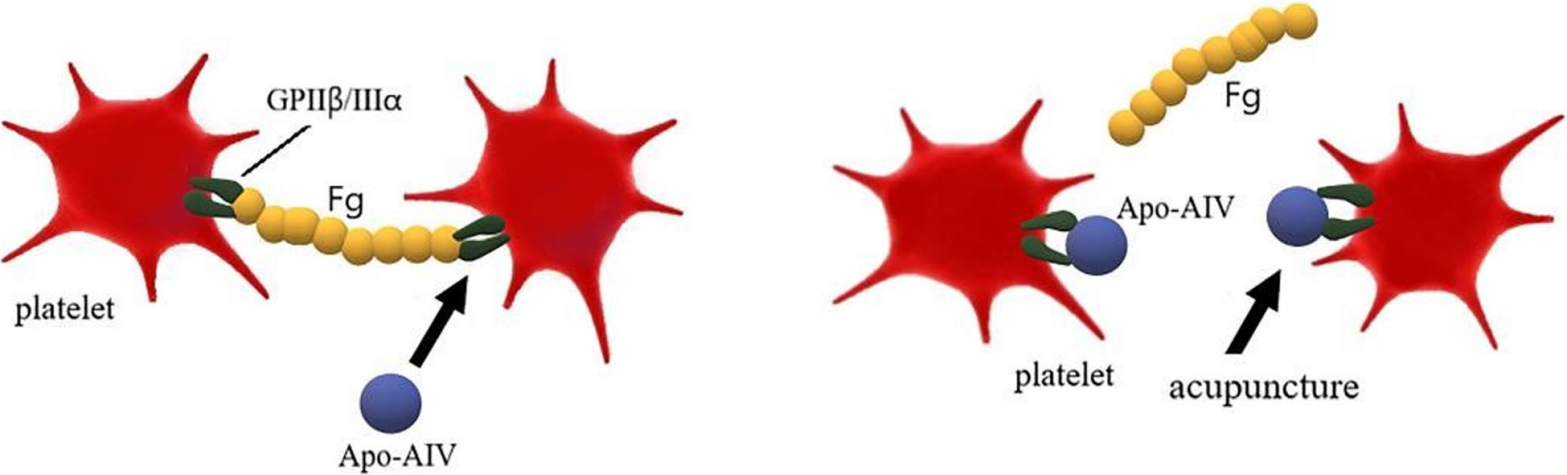
The potential influence that acupuncture works on Apo A-IV level. Acupuncture may antagonize the binding of platelets to fibrinogen by changing the level of apolipoprotein A-IV, thereby reducing the PAR in blood and reducing the formation of plaque

In terms of coagulation-related indicators, most scientists believe that inflammation can stimulate the vascular endothelium to activate platelets and promote the binding of platelets to fibrinogen, thereby promoting arteriosclerotic thrombosis. Our previous study revealed that apolipoprotein A-IV is a new ligand for platelet GPIIβ/IIIα integrin, which competitively inhibits the binding of glycoprotein GPIIβ/IIIα to fibrinogen and inhibits platelet aggregation [27, 29]. In this study, platelet function will be used as an entry point to explore whether acupuncture can antagonize the binding of platelets to fibrinogen by changing the level of apolipoprotein A-IV, thereby reducing the PAR in blood and reducing the formation of plaque. Figure 5 shows the way in which acupuncture may affect apolipoprotein A-IV.

### Selection of medication and placebo acupuncture method

There are different methods for the sham group based on acupuncture research, such as superficial skin acupuncture, non-acupoint acupuncture, and random acupoint acupuncture. According to the purpose of this study, we will adopt blunt needle acupuncture, which can avoid the effect of acupuncture epidermis therapy, and the design is more rigorous.

For drug selection, this study will use aspirin plus statins. The US 2014 AHA/ASA first-line prevention guidelines for stroke recommended that patients with asymptomatic carotid stenosis take aspirin or statin drugs [30]. Previous studies have suggested that the recommended dose of long-term aspirin is 75–150 mg/day. Most participants in this study are mild symptomatic carotid atherosclerotic plaque patients, which is consistent with the primary prevention of stroke. Based on the opinions of the Ethics Committee of the Guangdong Provincial Hospital of Traditional Chinese Medicine and patient’s risk factors, the dose of aspirin was adjusted to 100 mg/day. Statins exert beneficial effects for arteriosclerosis patients by reducing blood lipids and stabilizing plaques. According to the systematic analysis of the effects of different doses of atorvastatin on Chinese carotid artery stenosis (CAS) patients, patients taking 10 mg/day suffered fewer side effects, such as transverse muscle dissolution and elevated transaminase, while the treatment effect showed no significant difference. From an ethical point of view, the clinical dose of atorvastatin used in this trial is 10 mg/day after discussion.

### The basis of treatment sequence selection

Referring to the prior related research and taking into account patient compliance and other factors [31–33], the course of treatment is set at 12 weeks with 2 treatments per week for 24 treatments in total. Participants must complete at least 80% of treatments to be considered compliant. Patients will be followed up for 12 weeks after finishing treatment.

### Limitations of the study

During clinical implementation, this trial is limited by human resources and funding restrictions; thus, the detected timepoints and the numbers of cases are still not enough to meet the requirements. In terms of outcome indicators, the influence of diet or detection errors on lipid levels cannot be ignored. Although the trial emphasizes evaluating the efficacy of acupuncture for cervical atherosclerosis plaques, there is still insufficient evidence for Apo A-IV testing.

The elimination of plaques is affected by the structure of plaques themselves to a great extent. Subsequent studies will be conducted to quantify the composition and structure of plaques to objectively evaluate the therapeutic effect and treatment degree of acupuncture on different types of cervical atherosclerotic plaques.

## Data Availability

The participant-level dataset and statistical code will be available after the study is complete by asking for the consent or approval of Wenbin Fu (principal investigator). The results will be communicated to main investigators and sponsors and other relevant groups via publications; reporting results will be saved in ResMan Research Manager of the Clinical Trial Management Public Platform.

## Abbreviations

BMI: Body mass index
CAD: Carotid atherosclerosis disease
CAS: Carotid artery stenosis
CI: Confidence interval
CRF: Common reporting format
CVD: Cardiovascular disease
TA: The acupuncture group
MC: The medication group
SA: The sham acupuncture group
VPQ: Vessel plaque quantification
PV: The total plaque volume
GSM: The grey-scale median
IMT: Intima-media thickness
Apo A-IV: Apolipoprotein A-IV level
PLT: Platelet count
FIB: Fibrinogen
PAR: Platelet aggregation rate
PP: Per-protocol
ITT: Intention-to-treat
MI: Myocardial infarction
LDL: Low-density lipoprotein
TG: Triglyceride
HDL: High-density lipoprotein
SD: Standard deviation
